# Tetracycline and multidrug resistance in the oral microbiota: differences between healthy subjects and patients with periodontitis in Spain

**DOI:** 10.1080/20002297.2020.1847431

**Published:** 2020-11-23

**Authors:** Alexandre Arredondo, Vanessa Blanc, Carolina Mor, José Nart, Rubén León

**Affiliations:** a Department of Microbiology, Dentaid Research Center, Cerdanyola Del Vallès, Spain; b Departament De Genètica I Microbiologia, Universitat Autònoma De Barcelona, Bellaterra, Spain; c Department of Periodontology, Universitat Internacional De Catalunya, Barcelona, Spain

**Keywords:** Antibiotic resistance, tetracycline, periodontitis, subgingival microbiota, multidrug resistance

## Abstract

**Introduction**: Antibiotic resistance is widely found even among bacterial populations not having been exposed to selective pressure by antibiotics, such as tetracycline. In this study we analyzed the tetracycline-resistant subgingival microbiota of healthy subjects and of patients with periodontitis, comparing the prevalence of *tet* genes and their multidrug resistance profiles.

**Methods**: Samples from 259 volunteers were analyzed, obtaining 813 tetracycline-resistant isolates. The prevalence of 12 antibiotic resistance genes was assessed, and multidrug profiles were built. Each isolate was identified by 16S rRNA sequencing. Differences in qualitative data and quantitative data were evaluated using the chi-square test and the Mann-Whitney-U test, respectively.

**Results**: *tet*(M) was the most frequently detected *tet* gene (52.03%). We observed significant differences between the prevalence of *tet*(M), *tet*(W), *tet*(O), *tet*(32) and *tet*(L) in both populations studied. Multidrug resistance was largely observed, with resistance to kanamycin being the most detected (83.64%). There were significant differences between the populations in the prevalence of kanamycin, chloramphenicol, and cefotaxime resistance. Resistant isolates showed significantly different prevalence between the two studied groups.

**Conclusion**: The high prevalence of multidrug resistance and tetracycline resistance genes found in the subgingival microbiota, highlights the importance of performing wider and more in-depth analysis of antibiotic resistance in the oral microbiota.

## Introduction

Antibiotics have been used in clinical practice since their discovery, saving countless lives. Tetracycline was one of the first antibiotics to be discovered, in the 1940s, and its broad-spectrum activity and relatively few side effects made it a widely used antibiotic [1]. Its appealing properties led to extensive use, exerting a great deal of selective pressure on bacteria, which did not take long to become resistant [2]. Despite the rise of resistance, tetracyclines were and still are used in the treatment of some human infections [3], and especially in the cattle industry as growth promoters due to their anti-inflammatory effects on the gastrointestinal tract of the animals [4]. It has been reported that the use of antimicrobials in the cattle industry has an impact on the microbiota that lives in soils or sediments where all the wastes of such industry are left, increasing the prevalence of antimicrobial resistance genes in bacterial populations that had previously had little or no contact with antibiotics [5]. This abuse and misuse of antimicrobials may have repercussions on human health, increasing people’s chances of carrying antimicrobial-resistant bacteria without having been exposed to the antimicrobials in question. Such is the case of the oral environment, in which bacteria carrying tetracycline resistance genes have been previously detected [6,7]. Antibiotics have been used as adjuvants in certain dental treatments such as the treatment of periodontitis, which has been reported to benefit from the action of antimicrobials [8,9]. Currently, the main antibiotics used in periodontal treatment are amoxicillin and metronidazole [10], however, tetracyclines have been widely used in the past and there are reports of tetracycline resistance in the oral microbiota [7,11].

Resistance to tetracycline can be expressed through efflux pumps, ribosomal protection proteins (RPPs) and inactivation enzymes. Tetracycline resistance genes code for these mechanisms and are widespread among oral bacteria, where *tet*(M) is the most common [1]. Other genes coding for RPPs have been described in the oral environment, such as *tet*(Q), *tet*(O), *tet*(S) and *tet*(W), and genes that code for efflux pumps such as *tet*(B), *tet*(32), *tet*(K) and *tet*(L) can also be found within the oral microbiota [1]. Several genes, including *tet*(37) and *tet*(X), have been described as genes coding for inactivating enzymes, but so far only *tet*(37) has been found in the oral environment [12,13].

Furthermore, the strong presence of Tn*916/1545* family transposons in the oral microbiota, which can carry tetracycline, macrolide and/or aminoglycoside resistance genes, among others [14], means that those tetracycline-resistant microorganisms may also be resistant to other antimicrobials. Multidrug resistance is an increasingly troubling issue for the health authorities [15], and although multiple efforts are being made to provide more information, little is known regarding multidrug resistance in the oral environment.

It has been widely described that the subgingival microbiota of patients with periodontitis differs from that of healthy subjects [16] and therefore, the prevalence of antimicrobial resistance genes and the prevalence of multidrug resistance among these bacterial populations could be different.

The use of antibiotics from the past to treat infections caused by antibiotic-resistant bacteria has been proposed, on the grounds that due to the lack of selective pressure, the characteristics that once conferred resistance might have disappeared [17]. This might be the case with tetracyclines, whose use has declined over the years. There is limited knowledge regarding the distribution of tetracycline resistance in the subgingival microbiota, and the few papers that address this topic are outdated or focus on either particular genes or specific microorganisms. Therefore, the objective of this study was to analyze the prevalence and distribution of 11 *tet* genes and the multidrug resistance profiles of the tetracycline-resistant subgingival microbiota isolated from healthy volunteers and patients with periodontitis.

## Materials and methods

### Sample collection and culture

The samples included in this work were part of two previous studies [18,19], whose research protocols were approved by the Ethics Committee of the Universitat Internacional de Catalunya (UIC), (Barcelona, Spain) with study numbers: PER-ECL-2011-06-NF and ODO-2014-01. Both research protocols complied with the principles of the Declaration of Helsinki. Subgingival samples were taken from 259 volunteers including 129 periodontally healthy subjects and 130 subjects with periodontitis. Samples were obtained at the Department of Periodontology of the UIC. All volunteers signed an Institutional review board-approved informed consent form. Patients with periodontitis were diagnosed with generalized severe chronic periodontitis [20] or stage III or IV generalized grade B or C periodontitis [21]. To be included in the study, periodontally healthy subjects had to have at least six teeth per quadrant, probing depths ≤ 3 mm and absence of moderate or severe gingivitis. Smokers of more than five cigarettes per day, wearers of orthodontic appliances and pregnant or breastfeeding women were not included in the study. None of the volunteers took antibiotics or nonsteroidal anti-inflammatory drugs at least three months prior to the sampling, presented any systemic disease or took any chronic medication.

Subgingival samples were taken by placing two sterile paper points in the deepest site of each quadrant for 20 seconds and stored in 2 ml of reduced transport fluid without ethylenediaminetetraacetic acid (EDTA) [22]. Vials with the paper points were sent to the laboratory at 4°C to be processed within the same day. Subgingival samples were dispersed by vortex for 60 seconds. Serial dilutions of each sample were plated on blood agar plates (Blood agar base No. 2; Oxoid Ltd, Basingstoke, UK) containing 5% horse blood, hemin (5 mg/L) and menadione (1 mg/L) and on blood agar plates with and without 8 μg/ml tetracycline. All plates were incubated under anaerobic conditions (10% H_2_, 10% CO_2_ and 80% N_2_) at 37°C for 72 h. All of the morphologically different colonies were isolated and re-plated to obtain pure cultures that were preserved at −80°C in a 30% glycerol solution.

### In vitro *antibiotic resistance testing*

Resistance to six other antibiotics was tested using blood agar plates containing 1 μg/ml of erythromycin (ERY), 64 μg/ml of kanamycin (KAN), 8 μg/ml of chloramphenicol (CHL), 128 μg/ml of streptomycin (STR), 2 μg/ml of cefotaxime (CTX), and 8 μg/ml of amoxicillin (AMX) (all antimicrobials were obtained as pure powder from Sigma Aldrich, St. Louis, MO, USA). Antimicrobial breakpoint concentrations were set according to the recommendations of the Clinical and Laboratory Standards Institute (CLSI) [23] and the European Committee on Antimicrobial Susceptibility Testing (EUCAST) [24]. However, most of the species present in the oral environment are not covered by any of these organizations. Therefore, antibiotic concentrations were chosen based on taxonomic relatedness to oral bacteria, using the higher concentration of antibiotics when in doubt (Table S1). Incubation was performed at 37°C under anaerobic conditions for 72 h.

### DNA extraction

DNA extraction was performed on each isolate using the QIAamp DNA Mini Kit (Qiagen, Hilden, Germany) following the manufacturer’s instructions with some modifications. Cells were suspended in 180 μl of a 20 mg/ml lysozyme solution (20 mM Tris-Hcl, pH 8.0, 2 mM EDTA, 1.2% Triton X-100) and incubated for 30 min at 36°C. Then, 200 μl of Buffer AL (provided in the kit), 10 μl of RNase A (20 mg/ml) and 10 μl of proteinase K (20 mg/ml) were added and incubated for 30 min at 56°C. Further steps were conducted according to the manufacturer’s protocol. Lastly, DNA was re-suspended in 100 μl of buffer AE (provided in the kit), visualized in a 0.5% agarose gel and quantified using a Nanodrop 2000 C UV-vis spectrophotometer (Nanodrop Technologies, Wilmington, DE, USA).

### *Sequencing of the* 16S rRNA *gene*

The *16S rRNA* gene was amplified by the polymerase chain reaction (PCR) using universal primers 27 F and 1544 R (Table 1). PCR amplification was carried out applying a T3000 Thermocycler (Biometra, Goettingen, Germany) under the following conditions: 5 min at 95°C, followed by 35 cycles at 95°C for 60 sec, 57°C for 60 sec and 72°C for 60 sec; followed by another 10 min at 72°C. Sequencing of the gene *16S rRNA* was performed in Macrogen, Inc. (Amsterdam, The Netherlands). The sequences obtained were aligned to form a single contig and were identified by comparison with those available at the National Center for Biotechnology Information database (http://www.ncbi.nlm.nih.gov) using BLAST software (https://blast.ncbi.nlm.nih.gov/Blast.cgi). Only sequences with > 99% of similarity were accepted to identify isolates at species level and the 16S rRNA sequences were deposited in GenBank (accession numbers MT807114-MT807900).

**Table 1.** List of primers used in this study

Primers	Sequence 5ʹ-3’	Size (bp)	Tm	Tm Multiplex	Multiplex group	Ref.
tetMF tetMR	GCG TAC AAG CAC AGA CTC GT AGC CAT AGC GTA TCC CCT CC	1142	61	64	1	[6]
tetWF tetWR	GAG AGC CTG CTA TAT GCC AGC GGG CGT ATC CAC AAT GTT AAC	168	64			[57]
intF intR	GGC TAC AGA CCG AGT ACC AGC GGA ACT TGA CGT TCG CCA CT	684	61			[6]
ermBF ermBR	GGT AAA GGG CAT TTA ACG AC CGA TAT TCT CGA TTG ACC CA	494	55	60	2	[58]
tetQF tetQR	AGA ATC TGC TGT TTG CCA GTG CGG AGT GTC AAT GAT ATT GCA	167	50			[57]
tet32F tet32R	GAA CCA GAT GCT GCT CTT CAT AGC CAC GCC CAC ATG AT	620	57			[59]
tetLF tetLR	TCG TTA GCG TGC TGT CAT TC GTA TCC CAC CAA TGT AGC CG	267	55			[60]
tetOF tetOR	AAC TTA GGC ATT CTG GCT CAC TCC CAC TGT TCC ATA TCG TCA	515	55	58	3	[61]
tetSF tetSR	GAA AGC TTA CTA TAC AGT AGC AGG AGT ATC TAC AAT ATT TAC	168	50			[57]
tet31F tet31R	CAA TCA CGC CCA AAA GAA TGT GCC ATC CCA GTT TGT	564	53			[62]
tetBF tetBR	AAT AGC CAC TAA ATG GGG CG ATA ACA CCG GTT GCA TTG GT	243	58	56.5	4	[6]
tetKF tetKR	TCG ATA GGA ACA GCA GTA CAG CAG ATC CTA CTC CTT	169	52.8			[60]
tet37F tet37R	ATG GTT CGC TAT TAC TCT AAC ATC AGT CTC ATA TTT CGA CA	170	50			[6]
27 F 1544 R	GAG TTT GAT CCT GGC TCA G AGA AAG GAG GTG ATC CAG CC	approx. 1500	57	-	-	[63]

### Detection of antibiotic resistance genes

For the detection of antibiotic resistance genes, four multiplex-PCR reactions were performed using four sets of primers (Table 1) as previously described [6]. The amplified products were evaluated by electrophoresis using a 2% agarose gel. Some of these amplified products were sequenced (Macrogen, Inc., Amsterdam, The Netherlands), and used as positive controls. The GenBank accession numbers for the positive controls are available in the supplementary material.

### Statistical analyses

Qualitative data were obtained when screening for genes and when testing the ability of the isolates to grow in media containing a breakpoint antimicrobial concentration. Isolates were considered resistant to the antimicrobial if they were able to grow in the medium containing antibiotic and susceptible if they were not. To compare the prevalence of antibiotic resistance and of antibiotic resistance genes in both healthy subjects and subjects with periodontitis, data were analyzed using the chi-square test. To compare the bacterial loads resistant to tetracycline, the non-parametric Mann–Whitney U test was used. A nominal significance level of 5% (*p* < 0.05) was applied for both tests.

## Results

One hundred and twenty-nine subgingival samples from periodontally healthy subjects (SPHS) and 130 subgingival samples from subjects with periodontitis (SSP) were collected. SPHS were from patients between the ages of 19–24 (mean of 21.5 ± 3.3) years and SSP were from patients 24 to 82 (mean of 51.25 ± 11.97) years of age.

SSP grown on blood agar showed a mean bacterial load of 6.54 log_10_ colony-forming units per milliliter (cfu/ml) (± 0.91), while SPHS showed a mean bacterial load of 5.64 log_10_ cfu/ml (± 0.8). When grown on media with tetracycline, SSP showed a mean of 4.87 log_10_ cfu/ml (± 2.09), 2.14% of the total bacterial load, *versus* the 3.66 log_10_ cfu/ml (±1.63) for SPHS, 1.05% of the total bacterial load, which did not represent a significant difference (*p* = 0.76). Eighty-six-point eighty-two percent (86.82%) (n = 112) of SPHS and 86.15% (n = 112) of SSP harbored tetracycline-resistant bacteria, from which we obtained a total of 813 isolates, belonging 448 to SPHS and 365 to SSP.

*Streptococcus* sp. were the most frequently isolated species, representing 75.62% in SSP and 75.89% in SPHS. In both groups, *Streptococcus oralis* was the most frequently isolated species (n = 83), followed by other streptococcal species such as *Streptococcus mitis* (n = 81), *Streptococcus intermedius* (n = 79) and *Streptococcus constellatus* (n = 58). The most frequently isolated non-streptococcal species was *Prevotella intermedia* (n = 31) followed by *Prevotella nigrescens* (n = 25).

When comparing the prevalence of certain species between the two groups of subjects, significant differences were observed: *Gemella haemolysans* (5-fold, *p* = 0.016), *S. oralis* (3-fold, *p* < 0.01), *Streptococcus pneumoniae* (4-fold, *p* < 0.01) and *Streptococcus sanguinis* (4-fold, *p* < 0.01) were more prevalent in SPHS, while *Streptococcus anginosus* (2-fold, *p* = 0.036), *S. constellatus* (6-fold, *p* < 0.01), *Streptococcus gordonii* (2-fold, *p* = 0.022), *Streptococcus parasanguinis* (3-fold, *p* = 0.017) and *Streptococcus tigurinus* (7-fold, *p* = 0.049) were more prevalent in SSP (Table S2).

A list of the identified species of the tetracycline-resistant microorganisms isolated in this study, the prevalence of the antibiotic resistance genes screened and the multidrug resistance profile for the six antibiotics tested is displayed in Table 2. The genes *tet*(M) (52.03%), *tet*(32) (8.24%) and *tet*(O) (7.75%) were the most frequently detected *tet* genes. Significant differences were also observed in the prevalence of *tet*(M) (*p* < 0.01), *tet*(W) (*p* < 0.01), and *tet*(O) (*p* < 0.01), which were higher in SPHS, and *tet*(32) (*p* < 0.01) and *tet*(L) (*p* < 0.01), which were higher in SSP. The *intTn* gene, which codes for an integrase located in transposons of the family Tn*916/1545* was widely detected (79.58%), being more prevalent in SPHS (81.92%) than in SSP (76.71%).

**Table 2.** Species identified from SPHS and SSP and the number of isolates carrying each antimicrobial resistance gene and their phenotypical resistance. The asterisk indicates significant differences between SPHS and SSP isolates. AMX: amoxicillin, CTX: cefotaxime, CHL: chloramphenicol, STR: streptomycin, ERY: erythromycin, KAN: kanamycin

		Antimicrobial resistance genes														Antimicrobial resistance					
	Species	N	*intTn*	*tet*(W)	*tet*(O)	*tet*(M)	*tet* [31]	*tet* [32]	*tet*(B)	*tet*(L)	*tet*(S)	*tet*(Q)	*tet* [37]	*tet*(K)	*erm*(B)	AMX	CTX	CHL	STR	ERY	KAN
SSP	*Actinomyces* sp.	1	0	1	0	0	0	0	0	0	0	0	0	0	0	0	1	0	1	0	1
	*Bacteroides* sp.	2	0	0	0	0	0	0	0	0	0	2	0	0	0	0	2	0	2	2	2
	*Gemella* sp. *	2	2	0	0	2	0	0	0	0	0	0	0	0	0	1	0	0	1	2	2
	*Granulicatella* sp.	2	0	0	0	0	0	0	0	0	0	0	0	0	0	0	0	0	0	0	0
	*Lachnoanaerobaculum* sp.	3	2	0	0	0	0	0	0	0	0	0	0	0	2	1	2	0	2	2	2
	*Mogibacterium* sp.	1	0	1	0	0	0	1	0	0	0	0	0	0	0	0	1	0	0	1	1
	*Neisseria* sp.	2	0	0	0	0	0	0	0	0	0	0	0	0	0	0	2	0	0	2	0
	*Peptoniphilus* sp.	2	2	0	0	0	0	0	0	0	0	0	0	0	0	0	0	0	0	0	0
	*Peptostreptococcus* sp.	1	0	0	0	0	0	0	0	0	0	0	0	0	0	0	0	0	0	0	0
	*Prevotella* sp. *	48	16	0	0	3	0	18	7	0	0	27	0	2	3	28	36	2	15	24	42
	*Proteus* sp.	1	1	0	0	0	0	0	0	0	0	0	0	0	1	1	1	1	1	1	1
	*Pseudomonas* sp.	2	0	0	0	0	0	0	0	0	0	0	0	0	0	2	2	2	2	2	2
	*Rothia* sp.	2	1	0	0	0	0	0	0	0	0	0	0	0	0	0	0	0	1	2	2
	*Slackia* sp.	2	0	0	0	0	0	0	0	0	0	0	0	0	0	0	0	0	0	1	0
	*Staphylococcus* sp.	2	0	0	0	0	0	0	0	0	0	0	0	0	0	0	2	0	0	0	0
	*Streptococcus* sp.	276	248	0	18	116	0	31	0	12	0	3	0	0	128	20	56	29	48	188	271
	*Veillonella* sp.	6	6	0	0	5	0	0	0	0	0	0	0	0	2	1	4	0	4	5	2
	Not identified	10	2	0	0	2	0	1	1	0	0	1	0	0	1	1	2	1	1	3	4
SPHS	*Abiotrophia* sp.	5	5	0	0	2	0	0	0	0	0	0	0	0	4	0	0	1	0	5	2
	*Bacilus* sp.	1	1	0	0	1	0	0	0	0	0	0	0	0	1	0	0	0	0	1	1
	*Butyrivibrio* sp.	4	1	0	2	1	0	1	0	0	0	0	0	0	1	2	0	0	2	1	4
	*Eubacterium* sp.	3	2	1	0	3	0	1	1	0	0	0	0	0	2	0	0	0	1	3	3
	*Gemella* sp. *	13	13	1	0	8	0	0	1	0	0	1	0	0	0	1	1	4	0	5	4
	*Granulicatella* sp.	3	1	0	0	0	0	0	0	0	0	0	0	0	1	0	0	0	0	1	0
	*Haemophilus* sp.	4	2	1	0	2	0	1	2	0	0	0	0	0	3	0	0	0	0	4	1
	*Neisseria* sp.	2	0	0	0	2	0	0	0	0	0	0	0	0	2	0	0	0	1	2	0
	*Prevotella* sp. *	25	0	0	0	0	0	1	3	0	0	16	0	1	0	16	15	4	13	18	22
	*Rothia* sp.	4	1	3	0	0	0	0	0	0	0	0	0	0	0	0	0	0	0	1	2
	*Staphylococcus* sp.	5	1	0	0	0	0	0	0	0	0	0	0	3	4	3	3	2	3	4	0
	*Streptococcus* sp.	340	305	44	42	250	0	12	2	0	1	5	0	0	218	39	12	68	59	227	283
	*Veillonella* sp.	12	11	5	1	9	0	0	0	0	0	0	0	0	5	3	5	2	4	12	6
	Not identified	27	24	3	0	17	0	0	0	0	0	3	0	0	20	7	0	3	7	27	20

**Figure 1.**
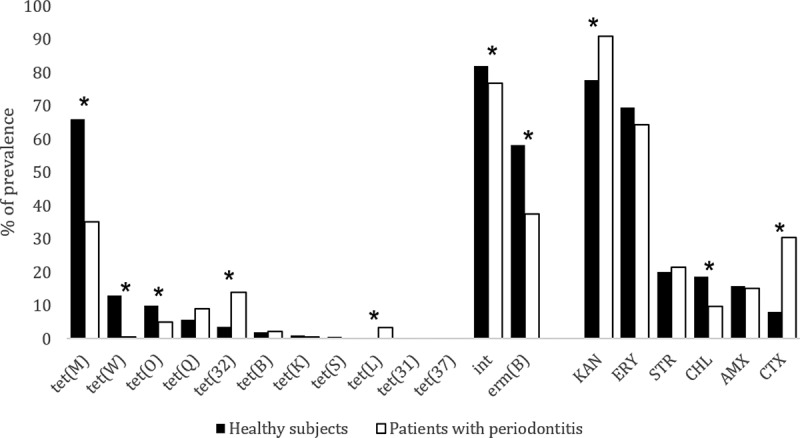
Prevalence (%) of *tet, int* and *erm*(B) genes and the prevalence (%) of subgingival isolates resistant to different antimicrobials (KAN: kanamycin, ERY: erythromycin, STR: streptomycin, CHL: Chloramphenicol, AMX: Amoxicillin, CTX: cefotaxime. The asterisk indicates significant differences between the isolates obtained from samples from periodontally healthy subjects and from samples from subjects with periodontitis

When assessing resistance to six different antimicrobials, a significant difference was found between the groups, where the SSP group showed three times more isolates. Significant differences were also observed between the isolates for susceptibility to all the antimicrobials, where SPHS isolates showed two times higher susceptibility. Moreover, we found that 91% of all the isolates were resistant to other antibiotics besides tetracycline (Table 3). Resistance to KAN was the most frequently observed (83.64%), followed by ERY (67.16%), STR (20.66%), CTX (18.08%), AMX (15.50%), and CHL (14.64%). Prevalence and significant differences of the studied genes and resistances between the two groups are shown in Figure 1.

**Table 3.** Number of isolates resistant to other antimicrobials. The asterisk indicates significant differences between samples from periodontally healthy subjects (SPHS) and the samples from subjects with periodontitis (SSP) isolates. In parentheses, the percentage of isolates over the total number of isolates from each population. Define meaning of asterisk

Number of other antimicrobials	SPHS isolates (%)		SSP isolates (%)		
0*	48	(10.71)		21	(5.75)
1	82	(18.30)		68	(18.63)
2	179	(39.96)		140	(38.36)
3	93	(20.76)		77	(21.10)
4	31	(6.92)		37	(10.14)
5	12	(2.68)		13	(3.56)
6*	3	(0.67)		9	(2.47)

All the identified species and their prevalence of the antibiotic resistance genes screened and the resistance to six antibiotics are presented in Table S2.

## Discussion

The present study investigated the tetracycline-resistant bacteria in subgingival samples of 129 periodontally healthy subjects and 130 patients with periodontitis, finding high loads of tetracycline-resistant bacteria, and a high prevalence of *tet* genes and multidrug resistance in both populations.

Bacterial counts, both total and that of resistant bacteria, were 10 times higher in SSP than in SPHS. This agrees with previous studies [25,26], in which the bacterial load in the periodontal pocket of patients with periodontitis was higher than that observed from periodontally healthy subjects. A higher percentage of tetracycline-resistant bacteria was also observed in SSP, probably due to the higher bacterial load of those samples.

Although the percentages of total tetracycline-resistant streptococci were similar in both groups of subjects, differences were observed at species level (Table S2). *S. sanguinis, S. oralis* and *S. pneumoniae*, which in our study were significantly more prevalent in SPHS, have been previously associated to commensal biofilms [27,28], while *S. anginosus, S. constellatus, S. gordonii, S. tigurinus* and *S. parasanguinis*, which in our study were significantly more prevalent in SSP, have been related to a periodontitis-associated microbiota [29–33]. However, the role of these species in periodontitis is not yet clear [34,35].

The prevalence of *G. haemolysans* was significantly higher in SPHS. This species has been described by metagenomic studies as an early colonizer of the oral biofilm [36,37] and therefore as part of the commensal microbiota. However, it has also been linked to opportunistic infections [38].

Isolates of the genus *Prevotella* were significantly more prevalent in SSP (2-fold). The increased richness of *Prevotella* species in SSP was responsible for this, increasing the total number of *Prevotella* isolates in SSP. However, *P. intermedia* and *P. nigrescens*, two *Prevotella* species that were present in both populations, did not show significantly different prevalences. The genus *Prevotella* has been linked to the progression of periodontitis, and therefore, a higher prevalence of this genus was expected in SSP. However, the increased richness of tetracycline-resistant *Prevotella* species observed in SSP is worth mentioning, since most reports have focused their attention on *P. intermedia* and *P. nigrescens* [39,40], and little is known about the prevalence of tetracycline resistance in other oral *Prevotella* species.

The prevalence of Gram-negative anaerobes was low, and species usually isolated from patients with periodontitis, such as *Porphyromonas gingivalis* or *Fusobacterium nucleatum*, were not detected in this study. This might be due to the high susceptibility that these organisms have to tetracycline, in most cases showing minimum inhibition concentrations lower than the 8 μg/ml tetracycline breakpoint concentration used in this study [41,42], which makes them unlikely to be isolated in such conditions, as seen in previous studies [7].

In this study, we found the genes *tet*(W), *tet*(O), *tet*(32), *tet*(B), *tet*(Q) and *tet*(K), distributed among the genera *Eubacterium, Gemella, Haemophilus, Veillonella, Butyrivibrio* and *Prevotella* which, according to the tetracycline resistance genes database available at http://faculty.washington.edu/marilynr/, had not been previously reported (Table 2). However, further studies are needed to confirm these results. For instance, the detection of the gene *tet*(B) in streptococci, as recently described [43], or new *tet* genes being described in the oral environment [44] show that there is still much to be unveiled regarding tetracycline resistance genes. The prevalence of the gene *tet*(M) among the SPHS in our study was similar (65.85%) to what has been previously described [7,45]. Nevertheless, the prevalence of this gene in SSP was much lower (35.07%) than what other authors found in Greek and US subjects with periodontitis [45,46], but similar to the percentages found in Dominican patients [6]. These differences might be explained by the geographical constraints of the populations involved in the studies or due to their methodological approaches, such as pooling the samples, the DNA or different selection criteria of the isolates. As previously discussed, the streptococcal species were significantly different between SPHS and SSP, which might be the cause for the differences in prevalence of *tet*(M) between the two groups studied. While 73.53% of the streptococci isolated from SPHS showed *tet*(M), only 42.02% of the streptococci isolated from SSP did, indicating that those streptococci associated to a healthy biofilm might be more susceptible to carry *tet*(M). In our study, the prevalence of *tet* [32] was higher in SSP, as previously described [6], which might be due to the increased prevalence of this gene in periodontitis-associated species such as *S. constellatus* and *P. intermedia*¸ although other authors have also detected this gene in some commensal bacteria [47]. The gene *tet*(W) can be frequently detected in the DNA from pooled saliva and plaque samples [7,48,49], but its distribution within the subgingival biofilm has only been studied by Collins et al. [6]. In that study, *tet*(W) was, on average, twice as prevalent in SSP than in SPHS. In our study, *tet*(W) was more ubiquitous in SPHS, since *S. intermedius* and *S. oralis*, which were more prevalent in SPHS, were some of the species that carried *tet*(W) most often.

Transposons of the Tn*916/1545* family are frequently found in the oral microbiota [50]. These transposons usually carry tetracycline resistance genes, and in some cases, genes that confer resistance to macrolides and/or aminoglycosides [51,52]. In order to estimate the presence of these transposons among the isolates, we used PCR to screen the *intTn* gene, which codes for an integrase located at the 3ʹ- ends of these transposons [14], the *tet*(M) gene, and the *erm*(B) gene, which confers resistance to macrolides and is often found in these conjugative elements [53]. The results showed a high prevalence of both genes in both groups, although they were significantly more prevalent in SPHS. The previously discussed differing prevalence of streptococcal species when comparing SPHS and SSP might be the reason for these differences, given that the streptococci of this study showed different profiles of antibiotic resistance genes. These results suggest a high prevalence of transposons of the Tn*916/1545* family in the tetracycline-resistant oral microbiota of healthy subjects and patients with periodontitis.

Most of the tetracycline-resistant isolates obtained in this study showed resistance to other antibiotics. Resistance to two antimicrobials besides tetracycline was the most common pattern, and a large part of this multidrug resistance was to KAN and ERY, which could be linked to the presence of the previously mentioned transposons [51]. Levels of multidrug resistance were high and similar in both populations, showing that the tetracycline-resistant subgingival microbiota is an important reservoir of antimicrobial resistance, which might be specially striking in subjects who have received little or no antibiotic therapy. The high prevalence of isolates resistant to antibiotics not commonly used, and therefore not exposed to selective pressure, might be due to the low fitness costs associated with the acquisition and maintenance of some mobile genetic elements that carry antibiotic resistance genes [54,55].

Significant differences were found when testing KAN and CHL resistance between both groups. The former was observed to be more prevalent in SSP isolates than in the SPHS isolates, where the contrary was observed for the latter. *Streptococcus* spp. and *Prevotella* spp. isolates might be accountable for these differences, since the *Prevotella* genus, which is known to show resistance to β-lactams and KAN [56], was twice as prevalent in SSP compared to SPHS. On the other hand, the differences in CHL resistance might be due to the unequal distribution of CHL resistance among streptococcal species, resulting in different percentages of this resistance between both groups (Table S2). The highest ratio of *P. intermedia* and *P. nigrescens* and the high prevalence of *S. constellatus* CTX-resistant in SSP were determinant for the higher prevalence of CTX resistance in SSP. Although the genus *Prevotella* is already known for its β-lactam resistance, high prevalence of CTX resistance among oral streptococci has not been previously reported; thus, it might pose a serious health issue, even more when considering their multidrug resistance capabilities.

While the differences found in this study were based on the periodontal diagnoses of the subjects, more certainty about the causes of the differences observed would most likely be achieved by including either the age or the sex of the subjects as variables of interest. Nonetheless, this study has shown high loads of bacteria exhibiting multidrug resistance and a variety of tetracycline resistance genes to be present in Spanish SSPs and SPHSs. However, significant differences were detected between the two groups in terms of i) bacterial species resistant to tetracycline; ii) prevalence of the screened genes and iii) multidrug resistance profiles. Moreover, the presence of some *tet* genes was detected in certain bacterial genera, which had not been previously described. Overall, we found that in Spanish subjects, although the use of tetracycline has been declining for many years, resistance to this antibiotic is still present in subgingival bacteria, which are a reservoir of tetracycline resistance genes and multidrug resistance, which, coupled with the high prevalence of conjugative transposons in the oral environment, might foster further increased spread of antimicrobial resistance.

## Supplementary Material

Supplemental Material
