# Supplementary material for: Tetracycline and multidrug resistance in the oral microbiota: differences between healthy subjects and patients with periodontitis in Spain
Source: J Oral Microbiol. 2020 Nov 23;13(1):1847431. doi: 10.1080/20002297.2020.1847431 (PMC7717685; doi:10.1080/20002297.2020.1847431)
Supplement: Supplemental Material [file ZJOM_A_1847431_SM3586.docx]

**Table S1.** List bacterial species and groups of species that were used in this study to set the antibiotic breakpoints.

| **Antibiotics** | **Species and/or groups of species** |
| --- | --- |
| **STR and KAN** | Enterococcus spp., Acinetobacter spp., Non-*Enterobacteriaceae*, Staphylococcus spp., *Enterobacteriaceae* and Pseudomonas aeruginosa |
| **CTX** | Streptococcus pneumoniae, viridans group streptococci, Haemophilus influenzae, Moraxella catarrhalis, Neisseria gonorrhoeae, Neisseria meningitidis, Pasteurella multocida, Kingella kingae and the "Non-species related breakpoints" group from EUCAST |
| **AMX** | *Enterobacteriaceae*, Enterococcus spp., Streptococcus pneumoniae, viridans group streptococci, Haemophilus influenzae, Moraxella catarrhalis, Burkholderia pseudomallei, and the "Non-species related breakpoints" group from EUCAST |
| **CHL** | Burkholderia pseudomallei, *Enterobacteriaceae*, Staphylococcus spp., Streptococcus groups A, B, C and G; *Streptococcus pneumoniae*, Haemophilus influenzae, Moraxella catarrhalis, Neisseria meningitidis, and the “Gram-positive anaerobes” group from CLSI |
| **ERY** | Streptococcus groups A, B, C and G; Streptococcus pneumoniae, Moraxella catarrhalis, Listeria monocytogenes and Kingella kingae |

Antibiotics should be written in full as a foot-note

**Table** **S2**. Species identified from SPHS and SSPD and the number of isolates carrying each antimicrobial resistance gene and their phenotypical resistance. The asterisk indicates significant differences between SPHS and SSPD isolates. AMX: amoxicillin, CTX: cefotaxime, CHL: chloramphenicol, STR: streptomycin, ERY: erythromycin, KAN: kanamycin. Continues in the next page.

|  | **Species** |  | **Antimicrobial resistance genes** | | | | | | | | | | | | | **Phenotypical resistance** | | | | | |
| --- | --- | --- | --- | --- | --- | --- | --- | --- | --- | --- | --- | --- | --- | --- | --- | --- | --- | --- | --- | --- | --- |
|  |  | **N** | ***intTn*** | ***tet(*W)** | ***tet*(O)** | ***tet*(M)** | ***tet*(31)** | ***tet*(32)** | ***tet*(B)** | ***tet*(L)** | ***tet*(S)** | ***tet*(Q)** | ***tet*(37)** | ***tet*(K)** | ***erm*(B)** | **AMX** | **CTX** | **CHL** | **STR** | **ERY** | **KAN** |
| **SSPD** | ***Actinomyces odontolyticus*** | 1 | 0 | 1 | 0 | 0 | 0 | 0 | 0 | 0 | 0 | 0 | 0 | 0 | 0 | 0 | 1 | 0 | 1 | 0 | 1 |
|  | ***Bacteroides pyogenes*** | 2 | 0 | 0 | 0 | 0 | 0 | 0 | 0 | 0 | 0 | 2 | 0 | 0 | 0 | 0 | 2 | 0 | 2 | 2 | 2 |
|  | ***Gemella haemolysans* *** | 2 | 2 | 0 | 0 | 2 | 0 | 0 | 0 | 0 | 0 | 0 | 0 | 0 | 0 | 1 | 0 | 0 | 1 | 2 | 2 |
|  | ***Granulicatella adiacens*** | 2 | 0 | 0 | 0 | 0 | 0 | 0 | 0 | 0 | 0 | 0 | 0 | 0 | 0 | 0 | 0 | 0 | 0 | 0 | 0 |
|  | ***Lachnoanaerobaculum saburreum*** | 2 | 1 | 0 | 0 | 0 | 0 | 0 | 0 | 0 | 0 | 0 | 0 | 0 | 1 | 1 | 2 | 0 | 2 | 2 | 2 |
|  | ***Lachnoanaerobaculum sp.*** | 1 | 1 | 0 | 0 | 0 | 0 | 0 | 0 | 0 | 0 | 0 | 0 | 0 | 1 | 0 | 0 | 0 | 0 | 0 | 0 |
|  | ***Mogibacterium pumilum*** | 1 | 0 | 1 | 0 | 0 | 0 | 1 | 0 | 0 | 0 | 0 | 0 | 0 | 0 | 0 | 1 | 0 | 0 | 1 | 1 |
|  | ***Neisseria subflava*** | 2 | 0 | 0 | 0 | 0 | 0 | 0 | 0 | 0 | 0 | 0 | 0 | 0 | 0 | 0 | 2 | 0 | 0 | 2 | 0 |
|  | ***Peptoniphilus lacrimalis*** | 2 | 2 | 0 | 0 | 0 | 0 | 0 | 0 | 0 | 0 | 0 | 0 | 0 | 0 | 0 | 0 | 0 | 0 | 0 | 0 |
|  | ***Peptostreptococcus stomatis*** | 1 | 0 | 0 | 0 | 0 | 0 | 0 | 0 | 0 | 0 | 0 | 0 | 0 | 0 | 0 | 0 | 0 | 0 | 0 | 0 |
|  | ***Prevotella baroniae*** | 1 | 0 | 0 | 0 | 0 | 0 | 1 | 0 | 0 | 0 | 0 | 0 | 0 | 0 | 0 | 0 | 0 | 0 | 0 | 1 |
|  | ***Prevotella buccae*** | 1 | 1 | 0 | 0 | 0 | 0 | 0 | 0 | 0 | 0 | 1 | 0 | 0 | 1 | 1 | 1 | 0 | 1 | 1 | 1 |
|  | ***Prevotella denticola*** | 6 | 3 | 0 | 0 | 2 | 0 | 0 | 0 | 0 | 0 | 6 | 0 | 0 | 0 | 6 | 6 | 0 | 1 | 6 | 6 |
|  | ***Prevotella disiens*** | 3 | 1 | 0 | 0 | 0 | 0 | 0 | 0 | 0 | 0 | 2 | 0 | 0 | 1 | 1 | 3 | 0 | 2 | 1 | 3 |
|  | ***Prevotella intermedia*** | 19 | 7 | 0 | 0 | 0 | 0 | 12 | 2 | 0 | 0 | 7 | 0 | 0 | 0 | 11 | 14 | 2 | 9 | 8 | 17 |
|  | ***Prevotella melaninogenica*** | 2 | 1 | 0 | 0 | 0 | 0 | 0 | 0 | 0 | 0 | 2 | 0 | 0 | 0 | 0 | 0 | 0 | 1 | 0 | 1 |
|  | ***Prevotella nigrescens*** | 16 | 3 | 0 | 0 | 1 | 0 | 5 | 5 | 0 | 0 | 9 | 0 | 2 | 1 | 9 | 12 | 0 | 1 | 8 | 13 |
|  | ***Proteus miriabilis*** | 1 | 1 | 0 | 0 | 0 | 0 | 0 | 0 | 0 | 0 | 0 | 0 | 0 | 1 | 1 | 1 | 1 | 1 | 1 | 1 |
|  | ***Pseudomonas aeruginosa*** | 2 | 0 | 0 | 0 | 0 | 0 | 0 | 0 | 0 | 0 | 0 | 0 | 0 | 0 | 2 | 2 | 2 | 2 | 2 | 2 |
|  | ***Rothia dentocariosa*** | 2 | 1 | 0 | 0 | 0 | 0 | 0 | 0 | 0 | 0 | 0 | 0 | 0 | 0 | 0 | 0 | 0 | 1 | 2 | 2 |
|  | ***Slackia exigua*** | 2 | 0 | 0 | 0 | 0 | 0 | 0 | 0 | 0 | 0 | 0 | 0 | 0 | 0 | 0 | 0 | 0 | 0 | 1 | 0 |
|  | ***Staphylococcus saprophyticus*** | 2 | 0 | 0 | 0 | 0 | 0 | 0 | 0 | 0 | 0 | 0 | 0 | 0 | 0 | 0 | 2 | 0 | 0 | 0 | 0 |
|  | ***Streptococcus sanguinis*** | 1 | 0 | 0 | 0 | 0 | 0 | 0 | 0 | 1 | 0 | 0 | 0 | 0 | 0 | 0 | 0 | 0 | 0 | 1 | 1 |
|  | ***Streptococcus anginosus* *** | 27 | 24 | 0 | 0 | 13 | 0 | 3 | 0 | 4 | 0 | 0 | 0 | 0 | 10 | 0 | 2 | 3 | 12 | 13 | 27 |
|  | ***Streptococcus australis*** | 2 | 0 | 0 | 1 | 0 | 0 | 0 | 0 | 0 | 0 | 0 | 0 | 0 | 0 | 1 | 1 | 0 | 0 | 2 | 2 |
|  | ***Streptococcus constellatus* *** | 48 | 44 | 0 | 2 | 15 | 0 | 10 | 0 | 0 | 0 | 0 | 0 | 0 | 27 | 6 | 24 | 5 | 6 | 28 | 48 |
|  | ***Streptococcus cristatus*** | 6 | 6 | 0 | 0 | 4 | 0 | 1 | 0 | 0 | 0 | 0 | 0 | 0 | 1 | 0 | 1 | 0 | 0 | 3 | 5 |
|  | ***Streptococcus dentisani*** | 1 | 1 | 0 | 0 | 1 | 0 | 0 | 0 | 0 | 0 | 0 | 0 | 0 | 1 | 0 | 0 | 0 | 0 | 1 | 1 |
|  | ***Streptococcus gordonii* *** | 26 | 25 | 0 | 0 | 18 | 0 | 2 | 0 | 3 | 0 | 0 | 0 | 0 | 8 | 0 | 1 | 1 | 1 | 14 | 26 |
|  | ***Streptococcus infantis*** | 2 | 2 | 0 | 0 | 0 | 0 | 0 | 0 | 0 | 0 | 0 | 0 | 0 | 1 | 0 | 0 | 0 | 0 | 2 | 2 |
|  | ***Streptococcus intermedius*** | 30 | 30 | 0 | 0 | 11 | 0 | 3 | 0 | 0 | 0 | 0 | 0 | 0 | 20 | 3 | 4 | 3 | 3 | 24 | 30 |
|  | ***Streptococcus mitis*** | 39 | 35 | 0 | 3 | 15 | 0 | 2 | 0 | 0 | 0 | 2 | 0 | 0 | 14 | 4 | 2 | 6 | 5 | 26 | 38 |
|  | ***Streptococcus oligofermentans*** | 1 | 1 | 0 | 0 | 1 | 0 | 0 | 0 | 0 | 0 | 0 | 0 | 0 | 0 | 0 | 1 | 0 | 0 | 1 | 0 |
|  | ***Streptococcus oralis* *** | 17 | 15 | 0 | 0 | 9 | 0 | 5 | 0 | 3 | 0 | 0 | 0 | 0 | 5 | 1 | 7 | 1 | 5 | 14 | 17 |
|  | ***Streptococcus parasanguinis* *** | 14 | 8 | 0 | 6 | 2 | 0 | 0 | 0 | 0 | 0 | 0 | 0 | 0 | 6 | 1 | 1 | 2 | 1 | 12 | 14 |
|  | ***Streptococcus pneumoniae* *** | 6 | 4 | 0 | 2 | 2 | 0 | 1 | 0 | 0 | 0 | 0 | 0 | 0 | 1 | 0 | 1 | 1 | 1 | 3 | 6 |
|  | ***Streptococcus pseudopneumoniae*** | 4 | 4 | 0 | 0 | 1 | 0 | 0 | 0 | 0 | 0 | 0 | 0 | 0 | 2 | 0 | 0 | 0 | 0 | 2 | 4 |
|  | ***Streptococcus rubneri*** | 2 | 2 | 0 | 0 | 2 | 0 | 0 | 0 | 0 | 0 | 0 | 0 | 0 | 1 | 0 | 1 | 0 | 1 | 2 | 2 |
|  | ***Streptococcus salivarius*** | 7 | 7 | 0 | 0 | 0 | 0 | 1 | 0 | 0 | 0 | 0 | 0 | 0 | 5 | 0 | 1 | 0 | 1 | 6 | 6 |
|  | ***Streptococcus sanguinis* *** | 7 | 7 | 0 | 2 | 2 | 0 | 0 | 0 | 0 | 0 | 0 | 0 | 0 | 6 | 2 | 3 | 1 | 1 | 7 | 7 |
|  | ***Streptococcus sp.*** | 30 | 27 | 0 | 2 | 18 | 0 | 3 | 0 | 0 | 0 | 1 | 0 | 0 | 16 | 2 | 5 | 6 | 11 | 23 | 29 |
|  | ***Streptococcus tigurinus* *** | 6 | 6 | 0 | 0 | 2 | 0 | 0 | 0 | 1 | 0 | 0 | 0 | 0 | 4 | 0 | 1 | 0 | 0 | 4 | 6 |
|  | ***Veillonella atypica*** | 1 | 1 | 0 | 0 | 1 | 0 | 0 | 0 | 0 | 0 | 0 | 0 | 0 | 0 | 1 | 1 | 0 | 0 | 1 | 0 |
|  | ***Veillonella caviae*** | 1 | 1 | 0 | 0 | 1 | 0 | 0 | 0 | 0 | 0 | 0 | 0 | 0 | 1 | 0 | 1 | 0 | 1 | 1 | 1 |
|  | ***Veillonella dispar*** | 1 | 1 | 0 | 0 | 1 | 0 | 0 | 0 | 0 | 0 | 0 | 0 | 0 | 0 | 0 | 1 | 0 | 1 | 1 | 0 |
|  | ***Veillonella parvula*** | 3 | 3 | 0 | 0 | 2 | 0 | 0 | 0 | 0 | 0 | 0 | 0 | 0 | 1 | 0 | 1 | 0 | 2 | 2 | 1 |
|  | **Not identified** | 10 | 2 | 0 | 0 | 2 | 0 | 1 | 1 | 0 | 0 | 1 | 0 | 0 | 1 | 1 | 2 | 1 | 1 | 3 | 4 |

|  | **Species** |  | **Antimicrobial resistance genes** | | | | | | | | | | | | | **Phenotypical resistance** | | | | | |
| --- | --- | --- | --- | --- | --- | --- | --- | --- | --- | --- | --- | --- | --- | --- | --- | --- | --- | --- | --- | --- | --- |
|  |  | **N** | ***intTn*** | ***tet*(W)** | ***tet*(O)** | ***tet*(M)** | ***tet*(31)** | ***tet*(32)** | ***tet*(B)** | ***tet*(L)** | ***tet*(S)** | ***tet*(Q)** | ***tet*(37)** | ***tet*(K)** | ***erm*(B)** | **AMX** | **CTX** | **CHL** | **STR** | **ERY** | **KAN** |
| **SPHS** | ***Abiotrophia defectiva*** | 5 | 5 | 0 | 0 | 2 | 0 | 0 | 0 | 0 | 0 | 0 | 0 | 0 | 4 | 0 | 0 | 1 | 0 | 5 | 2 |
|  | ***Bacillus cereus*** | 1 | 1 | 0 | 0 | 1 | 0 | 0 | 0 | 0 | 0 | 0 | 0 | 0 | 1 | 0 | 0 | 0 | 0 | 1 | 1 |
|  | ***Butyrivibrio* sp** | 4 | 1 | 0 | 2 | 1 | 0 | 1 | 0 | 0 | 0 | 0 | 0 | 0 | 1 | 2 | 0 | 0 | 2 | 1 | 4 |
|  | ***Eubacterium saburreum*** | 3 | 2 | 1 | 0 | 3 | 0 | 1 | 0 | 0 | 0 | 0 | 0 | 0 | 2 | 0 | 0 | 0 | 1 | 3 | 3 |
|  | ***Gemella haemolysans* *** | 13 | 13 | 1 | 0 | 8 | 0 | 0 | 0 | 0 | 0 | 1 | 0 | 0 | 0 | 1 | 1 | 4 | 0 | 5 | 4 |
|  | ***Granulicatella adiacens*** | 3 | 1 | 0 | 0 | 0 | 0 | 0 | 0 | 0 | 0 | 0 | 0 | 0 | 1 | 0 | 0 | 0 | 0 | 1 | 0 |
|  | ***Haemophilus parainfluenzae*** | 4 | 2 | 1 | 0 | 2 | 0 | 1 | 2 | 0 | 0 | 0 | 0 | 0 | 3 | 0 | 0 | 0 | 0 | 4 | 1 |
|  | ***Neisseria subflava*** | 2 | 0 | 1 | 0 | 2 | 0 | 0 | 0 | 0 | 0 | 0 | 0 | 0 | 2 | 0 | 0 | 0 | 1 | 2 | 0 |
|  | ***Prevotella bivia*** | 2 | 0 | 0 | 0 | 0 | 0 | 0 | 0 | 0 | 0 | 2 | 0 | 0 | 0 | 2 | 0 | 1 | 1 | 2 | 2 |
|  | ***Prevotella denticola*** | 2 | 0 | 0 | 0 | 0 | 0 | 0 | 0 | 0 | 0 | 2 | 0 | 0 | 0 | 0 | 0 | 2 | 0 | 2 | 2 |
|  | ***Prevotella intermedia*** | 12 | 0 | 0 | 0 | 0 | 0 | 0 | 0 | 0 | 0 | 5 | 0 | 1 | 0 | 11 | 9 | 1 | 9 | 7 | 11 |
|  | ***Prevotella nigrescens*** | 9 | 0 | 0 | 0 | 0 | 0 | 1 | 3 | 0 | 0 | 7 | 0 | 0 | 0 | 3 | 6 | 0 | 3 | 7 | 7 |
|  | ***Rothia dentocariosa*** | 4 | 1 | 3 | 0 | 0 | 0 | 0 | 0 | 0 | 0 | 0 | 0 | 0 | 0 | 0 | 0 | 0 | 0 | 1 | 2 |
|  | ***Staphylococcus epidermidis*** | 5 | 1 | 0 | 0 | 0 | 0 | 0 | 0 | 0 | 0 | 0 | 0 | 3 | 4 | 3 | 3 | 2 | 3 | 4 | 0 |
|  | ***Streptococcus anginosus* *** | 17 | 15 | 1 | 4 | 10 | 0 | 3 | 0 | 0 | 0 | 0 | 0 | 0 | 14 | 1 | 0 | 9 | 6 | 14 | 17 |
|  | ***Streptococcus australis*** | 5 | 3 | 0 | 1 | 2 | 0 | 0 | 0 | 0 | 0 | 0 | 0 | 0 | 3 | 1 | 2 | 0 | 0 | 5 | 4 |
|  | ***Streptococcus constellatus* *** | 10 | 10 | 0 | 0 | 7 | 0 | 3 | 0 | 0 | 0 | 0 | 0 | 0 | 7 | 0 | 0 | 8 | 2 | 8 | 6 |
|  | ***Streptococcus cristatus*** | 10 | 9 | 0 | 1 | 7 | 0 | 1 | 0 | 0 | 0 | 0 | 0 | 0 | 7 | 1 | 1 | 1 | 0 | 5 | 5 |
|  | ***Streptococcus gordonii* *** | 15 | 15 | 4 | 0 | 12 | 0 | 1 | 0 | 0 | 0 | 1 | 0 | 0 | 9 | 1 | 1 | 5 | 4 | 9 | 12 |
|  | ***Streptococcus infantis*** | 4 | 3 | 0 | 1 | 2 | 0 | 0 | 0 | 0 | 0 | 0 | 0 | 0 | 2 | 0 | 0 | 0 | 1 | 2 | 3 |
|  | ***Streptococcus intermedius*** | 49 | 48 | 12 | 10 | 39 | 0 | 1 | 0 | 0 | 0 | 0 | 0 | 0 | 40 | 7 | 0 | 13 | 8 | 38 | 38 |
|  | ***Streptococcus massiliensis*** | 1 | 1 | 0 | 0 | 1 | 0 | 0 | 0 | 0 | 0 | 0 | 0 | 0 | 1 | 0 | 0 | 0 | 1 | 1 | 1 |
|  | ***Streptococcus mitis*** | 42 | 34 | 7 | 4 | 30 | 0 | 1 | 0 | 0 | 0 | 2 | 0 | 0 | 19 | 6 | 4 | 6 | 6 | 23 | 32 |
|  | ***Streptococcus oralis* *** | 66 | 61 | 7 | 9 | 54 | 0 | 1 | 2 | 0 | 0 | 0 | 0 | 0 | 41 | 3 | 0 | 7 | 17 | 40 | 61 |
|  | ***Streptococcus parasanguinis* *** | 5 | 4 | 1 | 2 | 4 | 0 | 0 | 0 | 0 | 0 | 0 | 0 | 0 | 2 | 0 | 0 | 1 | 0 | 2 | 3 |
|  | ***Streptococcus pneumoniae* *** | 34 | 26 | 1 | 4 | 25 | 0 | 1 | 0 | 0 | 0 | 0 | 0 | 0 | 15 | 7 | 0 | 3 | 8 | 17 | 26 |
|  | ***Streptococcus pseudopneumoniae*** | 18 | 14 | 4 | 4 | 10 | 0 | 0 | 0 | 0 | 0 | 1 | 0 | 0 | 7 | 3 | 0 | 3 | 1 | 13 | 18 |
|  | ***Streptococcus salivarius*** | 9 | 9 | 2 | 0 | 4 | 0 | 0 | 0 | 0 | 1 | 0 | 0 | 0 | 9 | 2 | 0 | 2 | 0 | 8 | 9 |
|  | ***Streptococcus sanguinis* *** | 35 | 35 | 5 | 0 | 26 | 0 | 0 | 0 | 0 | 0 | 0 | 0 | 0 | 28 | 6 | 4 | 9 | 3 | 27 | 30 |
|  | ***Streptococcus sinensis*** | 5 | 3 | 0 | 2 | 3 | 0 | 0 | 0 | 0 | 0 | 0 | 0 | 0 | 1 | 0 | 0 | 1 | 1 | 3 | 3 |
|  | ***Streptococcus tigurinus* *** | 1 | 1 | 0 | 0 | 1 | 0 | 0 | 0 | 0 | 0 | 0 | 0 | 0 | 0 | 0 | 0 | 0 | 0 | 0 | 1 |
|  | ***Streptococcus* sp** | 14 | 14 | 0 | 0 | 13 | 0 | 0 | 0 | 0 | 0 | 1 | 0 | 0 | 13 | 1 | 0 | 0 | 1 | 12 | 14 |
|  | ***Veillonella parvula*** | 4 | 4 | 2 | 1 | 4 | 0 | 0 | 0 | 0 | 0 | 0 | 0 | 0 | 2 | 1 | 0 | 1 | 1 | 4 | 2 |
|  | ***Veillonella ratti*** | 2 | 2 | 0 | 0 | 2 | 0 | 0 | 0 | 0 | 0 | 0 | 0 | 0 | 0 | 0 | 2 | 0 | 1 | 2 | 1 |
|  | ***Veillonella rogosae*** | 1 | 1 | 0 | 0 | 1 | 0 | 0 | 0 | 0 | 0 | 0 | 0 | 0 | 1 | 1 | 0 | 0 | 0 | 1 | 1 |
|  | ***Veillonella* sp** | 5 | 4 | 3 | 0 | 2 | 0 | 0 | 0 | 0 | 0 | 0 | 0 | 0 | 2 | 1 | 3 | 1 | 2 | 5 | 2 |
|  | **No determinado** | 27 | 24 | 3 | 0 | 17 | 0 | 0 | 0 | 0 | 0 | 3 | 0 | 0 | 20 | 7 | 0 | 3 | 7 | 27 | 20 |

**List of GenBank’s accession numbers:**

**16S sequences:**

MT807114, MT807115, MT807116, MT807117, MT807118, MT807119, MT807120, MT807121, MT807122, MT807123, MT807124, MT807125, MT807126, MT807127, MT807128, MT807129, MT807130, MT807131, MT807132, MT807133, MT807134, MT807135, MT807136, MT807137, MT807138, MT807139, MT807140, MT807141, MT807142, MT807143, MT807144, MT807145, MT807146, MT807147, MT807148, MT807149, MT807150, MT807151, MT807152, MT807153, MT807154, MT807155, MT807156, MT807157, MT807158, MT807159, MT807160, MT807161, MT807162, MT807163, MT807164, MT807165, MT807166, MT807167, MT807168, MT807169, MT807170, MT807171, MT807172, MT807173, MT807174, MT807175, MT807176, MT807177, MT807178, MT807179, MT807180, MT807181, MT807182, MT807183, MT807184, MT807185, MT807186, MT807187, MT807188, MT807189, MT807190, MT807191, MT807192, MT807193, MT807194, MT807195, MT807196, MT807197, MT807198, MT807199, MT807200, MT807201, MT807202, MT807203, MT807204, MT807205, MT807206, MT807207, MT807208, MT807209, MT807210, MT807211, MT807212, MT807213, MT807214, MT807215, MT807216, MT807217, MT807218, MT807219, MT807220, MT807221, MT807222, MT807223, MT807224, MT807225, MT807226, MT807227, MT807228, MT807229, MT807230, MT807231, MT807232, MT807233, MT807234, MT807235, MT807236, MT807237, MT807238, MT807239, MT807240, MT807241, MT807242, MT807243, MT807244, MT807245, MT807246, MT807247, MT807248, MT807249, MT807250, MT807251, MT807252, MT807253, MT807254, MT807255, MT807256, MT807257, MT807258, MT807259, MT807260, MT807261, MT807262, MT807263, MT807264, MT807265, MT807266, MT807267, MT807268, MT807269, MT807270, MT807271, MT807272, MT807273, MT807274, MT807275, MT807276, MT807277, MT807278, MT807279, MT807280, MT807281, MT807282, MT807283, MT807284, MT807285, MT807286, MT807287, MT807288, MT807289, MT807290, MT807291, MT807292, MT807293, MT807294, MT807295, MT807296, MT807297, MT807298, MT807299, MT807300, MT807301, MT807302, MT807303, MT807304, MT807305, MT807306, MT807307, MT807308, MT807309, MT807310, MT807311, MT807312, MT807313, MT807314, MT807315, MT807316, MT807317, MT807318, MT807319, MT807320, MT807321, MT807322, MT807323, MT807324, MT807325, MT807326, MT807327, MT807328, MT807329, MT807330, MT807331, MT807332, MT807333, MT807334, MT807335, MT807336, MT807337, MT807338, MT807339, MT807340, MT807341, MT807342, MT807343, MT807344, MT807345, MT807346, MT807347, MT807348, MT807349, MT807350, MT807351, MT807352, MT807353, MT807354, MT807355, MT807356, MT807357, MT807358, MT807359, MT807360, MT807361, MT807362, MT807363, MT807364, MT807365, MT807366, MT807367, MT807368, MT807369, MT807370, MT807371, MT807372, MT807373, MT807374, MT807375, MT807376, MT807377, MT807378, MT807379, MT807380, MT807381, MT807382, MT807383, MT807384, MT807385, MT807386, MT807387, MT807388, MT807389, MT807390, MT807391, MT807392, MT807393, MT807394, MT807395, MT807396, MT807397, MT807398, MT807399, MT807400, MT807401, MT807402, MT807403, MT807404, MT807405, MT807406, MT807407, MT807408, MT807409, MT807410, MT807411, MT807412, MT807413, MT807414, MT807415, MT807416, MT807417, MT807418, MT807419, MT807420, MT807421, MT807422, MT807423, MT807424, MT807425, MT807426, MT807427, MT807428, MT807429, MT807430, MT807431, MT807432, MT807433, MT807434, MT807435, MT807436, MT807437, MT807438, MT807439, MT807440, MT807441, MT807442, MT807443, MT807444, MT807445, MT807446, MT807447, MT807448, MT807449, MT807450, MT807451, MT807452, MT807453, MT807454, MT807455, MT807456, MT807457, MT807458, MT807459, MT807460, MT807461, MT807462, MT807463, MT807464, MT807465, MT807466, MT807467, MT807468, MT807469, MT807470, MT807471, MT807472, MT807473, MT807474, MT807475, MT807476, MT807477, MT807478, MT807479, MT807480, MT807481, MT807482, MT807483, MT807484, MT807485, MT807486, MT807487, MT807488, MT807489, MT807490, MT807491, MT807492, MT807493, MT807494, MT807495, MT807496, MT807497, MT807498, MT807499, MT807500, MT807501, MT807502, MT807503, MT807504, MT807505, MT807506, MT807507, MT807508, MT807509, MT807510, MT807511, MT807512, MT807513, MT807514, MT807515, MT807516, MT807517, MT807518, MT807519, MT807520, MT807521, MT807522, MT807523, MT807524, MT807525, MT807526, MT807527, MT807528, MT807529, MT807530, MT807531, MT807532, MT807533, MT807534, MT807535, MT807536, MT807537, MT807538, MT807539, MT807540, MT807541, MT807542, MT807543, MT807544, MT807545, MT807546, MT807547, MT807548, MT807549, MT807550, MT807551, MT807552, MT807553, MT807554, MT807555, MT807556, MT807557, MT807558, MT807559, MT807560, MT807561, MT807562, MT807563, MT807564, MT807565, MT807566, MT807567, MT807568, MT807569, MT807570, MT807571, MT807572, MT807573, MT807574, MT807575, MT807576, MT807577, MT807578, MT807579, MT807580, MT807581, MT807582, MT807583, MT807584, MT807585, MT807586, MT807587, MT807588, MT807589, MT807590, MT807591, MT807592, MT807593, MT807594, MT807595, MT807596, MT807597, MT807598, MT807599, MT807600, MT807601, MT807602, MT807603, MT807604, MT807605, MT807606, MT807607, MT807608, MT807609, MT807610, MT807611, MT807612, MT807613, MT807614, MT807615, MT807616, MT807617, MT807618, MT807619, MT807620, MT807621, MT807622, MT807623, MT807624, MT807625, MT807626, MT807627, MT807628, MT807629, MT807630, MT807631, MT807632, MT807633, MT807634, MT807635, MT807636, MT807637, MT807638, MT807639, MT807640, MT807641, MT807642, MT807643, MT807644, MT807645, MT807646, MT807647, MT807648, MT807649, MT807650, MT807651, MT807652, MT807653, MT807654, MT807655, MT807656, MT807657, MT807658, MT807659, MT807660, MT807661, MT807662, MT807663, MT807664, MT807665, MT807666, MT807667, MT807668, MT807669, MT807670, MT807671, MT807672, MT807673, MT807674, MT807675, MT807676, MT807677, MT807678, MT807679, MT807680, MT807681, MT807682, MT807683, MT807684, MT807685, MT807686, MT807687, MT807688, MT807689, MT807690, MT807691, MT807692, MT807693, MT807694, MT807695, MT807696, MT807697, MT807698, MT807699, MT807700, MT807701, MT807702, MT807703, MT807704, MT807705, MT807706, MT807707, MT807708, MT807709, MT807710, MT807711, MT807712, MT807713, MT807714, MT807715, MT807716, MT807717, MT807718, MT807719, MT807720, MT807721, MT807722, MT807723, MT807724, MT807725, MT807726, MT807727, MT807728, MT807729, MT807730, MT807731, MT807732, MT807733, MT807734, MT807735, MT807736, MT807737, MT807738, MT807739, MT807740, MT807741, MT807742, MT807743, MT807744, MT807745, MT807746, MT807747, MT807748, MT807749, MT807750, MT807751, MT807752, MT807753, MT807754, MT807755, MT807756, MT807757, MT807758, MT807759, MT807760, MT807761, MT807762, MT807763, MT807764, MT807765, MT807766, MT807767, MT807768, MT807769, MT807770, MT807771, MT807772, MT807773, MT807774, MT807775, MT807776, MT807777, MT807778, MT807779, MT807780, MT807781, MT807782, MT807783, MT807784, MT807785, MT807786, MT807787, MT807788, MT807789, MT807790, MT807791, MT807792, MT807793, MT807794, MT807795, MT807796, MT807797, MT807798, MT807799, MT807800, MT807801, MT807802, MT807803, MT807804, MT807805, MT807806, MT807807, MT807808, MT807809, MT807810, MT807811, MT807812, MT807813, MT807814, MT807815, MT807816, MT807817, MT807818, MT807819, MT807820, MT807821, MT807822, MT807823, MT807824, MT807825, MT807826, MT807827, MT807828, MT807829, MT807830, MT807831, MT807832, MT807833, MT807834, MT807835, MT807836, MT807837, MT807838, MT807839, MT807840, MT807841, MT807842, MT807843, MT807844, MT807845, MT807846, MT807847, MT807848, MT807849, MT807850, MT807851, MT807852, MT807853, MT807854, MT807855, MT807856, MT807857, MT807858, MT807859, MT807860, MT807861, MT807862, MT807863, MT807864, MT807865, MT807866, MT807867, MT807868, MT807869, MT807870, MT807871, MT807872, MT807873, MT807874, MT807875, MT807876, MT807877, MT807878, MT807879, MT807880, MT807881, MT807882, MT807883, MT807884, MT807885, MT807886, MT807887, MT807888, MT807889, MT807890, MT807891, MT807892, MT807893, MT807894, MT807895, MT807896, MT807897, MT807898, MT807899, MT807900

**Tetracycline resistance genes:**

KX034799, KX034800, KX034801, KX078641, KX034803, KX034804, KX034802, MT834961, MT834962, MT834963, MT843580
